# The Activity of the Skin in the Absorption of Medicines

**Published:** 1867-11

**Authors:** 


### Miscellaneous.

### The Activity of the Skin in the Absorption of Medicines.

BY DR. ROUSSIN.

While it is abundantly proved that many articles of the materia medica, united with fatty matters, applied to the skin with a proper amount of friction, are absorbed, experiments with similar articles in a state of solution give different results. According to Laurès, and others, a man may sit for hours in a bath containing 200 to 300 grammes of iodide of potassium, without the urine showing the slightest traces of iodine; in the same way one may remain in a bath containing from 20 to 60 grammes of corrosive sublimate, without the slightest salivation being produced, while it would be occasioned by much smaller quantities rubbed into the skin. Finally, Magendie left a rather concentrated solution of strychnine in contact with the skin without causing the slightest spasm.

The experiments of Dr. Roussin confirm what was previously known on the subject. He remained from an hour to an hour and a half in a bath, containing from 450 to 500 grammes of iodide of potassium; in no instance, when the body was dried or the solution of the iodide washed off on coming out of the bath, could the slightest trace of iodine be discovered within twenty-four hours in the urine, or the saliva. On the other hand, when, on leaving the bath, the solution adhering to the body, was permitted to evaporate spontaneously, iodine showed itself soon after in the urine. In one of his experiments the author wet his arms with solution of the iodide and then permitted the solution to evaporate spontaneously; four hours afterward iodine was found in both the urine and saliva. The experiments show that the skin being unbroken, iodide of potassium is absorbed only when it is left in substance in contact with the skin. This becomes still more evident from the following experiments:—The author sprinkled the anterior part of his body from the neck to the abdomen with finely powdered iodide of potassium, rubbing it into the skin. The urine for the next twenty-four hours gave abundant evidence of iodine. The same evidence was given when the experimenter wore a shirt which, with the exception of the bosom, had been wet with a solution of iodide of potassium (10 per cent. in strength,) and then dried.

Many other substances behave in a manner similar to iodide of potassium, and in this way we can understand the numerous symptoms of poisoning which have been observed, when through the medium of the clothes, or in some similar manner, poisons come in immediate contact with the skin.

The cause of this passive relation of the skin to medicinal substances dissolved in water with which it comes in contact, arises from the fact that it is not possible for the water to enter the pores of the skin, on account of the fatty character of its surface. On the contrary, water is repelled rather than attracted, just as it is by capillary tubes with fatty walls.

Dr. R. shows that the skin is not in reality wet by watery solutions brought in contact with it; that is to say, the water does not extend in a continuous layer over it, but being repelled by the greasy surface, forms drops upon it. Even after soaping of the skin, the spreading out, the adhesion of the drops which fall upon it, is only apparent; the solution again gathers into drops as soon as the layer of soap, which permitted its adhesion to the skin, is removed. The same thing is observed when the skin is treated with ether. So soon as this is evaporated, the original relations between the skin and watery solutions placed upon it are renewed, because a constant secretion of fat takes place from it. On the other hand, when a piece of skin, taken from the cadaver, is soaped after the removal of the layer of soap, water wets it, spreading itself out, without being gathered into drops.

The absorption of fatty matters by the skin, finds its natural explanation in the laws of capilarity; they can, rubbed into the skin, pass easily through the capillary vessels, and with them the substances with which they are incorporated, provided only they are minutely enough divided.

In like manner we can explain the absorption of solid matters when placed upon the skin in a pulverulent form, and here become mixed with its fatty secretion.

On the contrary, glycerine, which behaves toward the skin in a manner similar to water, is not to be used as a vehicle for substances which we desire to introduce into the economy through that organ.—(Rec. des Mem. de Med. etc. Milit. 3d ser. xviii, p. 134, Feb. 1867, in Schmidt's Jahrbücher, No. 7, 1867.)

